# Efficacy of Bisphosphonates in Total Hip Arthroplasty Patients: Systematic Review and Meta-Analysis

**DOI:** 10.3390/biomedicines12081778

**Published:** 2024-08-06

**Authors:** Alberto Di Martino, Konstantinos Valtetsiotis, Valentino Rossomando, Matteo Brunello, Barbara Bordini, Claudio D’Agostino, Federico Ruta, Francesco Traina, Cesare Faldini

**Affiliations:** 1 1st Orthopaedic and Traumatologic Clinic, IRCCS Rizzoli Orthopedic Institute, Via G.C. Pupilli 1, 40136 Bologna, Italyvalentino.rossomando@ior.it (V.R.); matteo.brunello@ior.it (M.B.); caludio.dagostino@ior.it (C.D.); federico.ruta@ior.it (F.R.); cesare.faldini@ior.it (C.F.); 2 Department of Biomedical and Neuromotor Science-DIBINEM, University of Bologna, 40126 Bologna, Italy; francesco.traina@ior.it; 3 Medical Technology Laboratory, IRCCS Istituto Ortopedico Rizzoli, Via di Barbiano 1/10, 40136 Bologna, Italy; barbara.bordini@ior.it; 4 Chirurgia Protesica e dei Reimpianti d’Anca e di Ginocchio, Rizzoli Orthopedic Institute, Via G.C. Pupilli 1, 40136 Bologna, Italy

**Keywords:** periprosthetic osteolysis, total hip arthroplasty, bisphosphonates, Bony Mass Density, Gruen’s zones

## Abstract

The scientific literature suggests that, if periprosthetic osteolysis (PPO) is not treated, it may have a negative impact on the results of a total hip replacement and possibly result in failure. This systematic review aimed to determine the efficacy of using bisphosphonates preventatively to limit PPO after a total hip arthroplasty (THA). Methods: A systematic review and meta-analysis were conducted following the Preferred Reporting Items for Systematic Reviews and Meta-Analyses (PRISMA) guidelines. A PICOS template was developed to ensure a structured approach. A search for relevant studies was performed across four databases, including Pubmed, Scopus, Embase, and Cochrane. They were all last searched on March 1st and were assessed using the Cochrane risk of bias tool for randomised studies. Results: The final analysis included seven studies with a total of 126 study group participants and 144 control group participants. The studies looked at Bony Mass Density in terms of bone loss on Gruen’s femoral zones after THA in a bisphosphonate (treatment) and control group (placebo/no treatment). The analysis revealed a statistically significant difference (*p* < 0.05) in favour of the bisphosphonate group in many of the included studies at 6, 12, and 24 postoperative months. Conclusions: This systematic review and meta-analysis, using the most recent applicable studies, showed the efficacy of bisphosphonates in limiting periprosthetic osteolysis after THA in a period between 6 and 24 postoperative months. Future studies should focus increasing group sizes and collecting results beyond the 2-year mark.

## 1. Introduction

Total hip arthroplasties (THAs) have been practiced in some form for over a century, while modern designs resembling those used today for over half a century [1]. In most high-income countries, estimates suggest that the number of THAs will increase. This reflects the demographic trends of an increase in the ratio of geriatric population brackets and a higher life expectancy. In the US, the number of primary and revision THAs between 2020 and 2030 is expected to increase by about 50% [2]. In the UK, the number of THAs from now to 2035 is expected to more than double [3]. Other western countries such as Canada, Denmark, and Australia are also all expected to see increases, at varying degrees [4,5,6].

The most common causes of failure of hip THAs are: periprosthetic joint infection, fractures, aseptic loosening, and dislocation [7,8]. Osteolysis is involved in wear, aseptic loosening, and fractures and, as such, its prevention and management are important in reducing the risk of failure. In total, 20–30% of patients who receive an arthroplasty will develop symptoms of periprosthetic osteolysis (PPO) within 10 years post operation [9,10]. It has been established in THAs that osteolysis has a linear correlation with the wear of the polyethylene cup [11,12]. The propensity for osteolysis as the prosthesis wears is largely intervariable and an individual’s genetic background, such as their SNPs, may influence the extent of the inflammatory response [13]. On a molecular level, PPO begins with two different mechanisms resulting in the same outcome. The first is mechanical-related due to the movement of the prosthetic joint producing wear particles. The other mechanism is related to the corrosion of the prosthesis, which also produces particles. This is why PPO has often been called “particle disease” [14]. The size, material type, number, and site of these particles have downstream effects that lead to PPO. In a periprosthetic joint without PPO, there is a balance between particle production and their removal from the immune system. PPO occurs when this balance is broken, causing topical inflammation and increased bone resorption [14,15]. There are, however, conflicting opinions on if and how particles cause osteolysis [16], so the exact mechanisms remain unknown [14].

PPO has limited treatment options. Once established, PPO is thought to inevitably continue progressing, so at some point, a revision surgery would be required [17,18]. For patients with mild PPO symptoms or for those where a surgery is not currently possible, non-surgical treatment options may be used. This includes the topical usage of stem cell therapy and the pharmaceutical administration of either osteoporotic drugs, anabolic treatments, or targeted anti-inflammatory treatments [17]. One of the pharmacologic agent classes used to treat or prevent PPO are bisphosphonates. Bisphosphonates are structurally stable chemical derivatives of inorganic pyrophosphate (PPi). Early research from the 1960s showed that PPi may bind to hydroxyapatite crystals and block calcification. Bisphosphonates are integrated preferentially into regions of active bone remodelling, which is frequently observed in circumstances where there is an increased rate of skeletal turnover. Bisphosphonates are classified into two categories: nitrogenous or non-nitrogenous. Nitrogenous bisphosphonates alter osteoclast development, survival, and cytoskeletal dynamics. Non-nitrogenous bisphosphonates trigger osteoclast apoptosis [19,20]. These mechanisms are used in periprosthetic osteolysis to slow down or inhibit its progression. They are administered orally or through IV [19]. This meta-analysis included alendronate, etidronate, and zoledronic acid. The first two are administered orally, while the third is administered through IV. Etidronate is considered to be a first-generation non-nitrogenous bisphosphonate whose relative potency is 1. Alendronate is a second-generation nitrogenous bisphosphonate whose relative potency is 500. Zoledronic acid is a third-generation nitrogenous bisphosphonate. The latter is among the most powerful bisphosphonates and its relative potency is 10,000 [19].

Since there have not been any meta-analyses on their usage in a couple of years, we aimed to give an update on their efficacy. The question we aim to answer is what is the efficacy of preventative pharmaceutical treatment in limiting peri-implant osteolysis compared to placebo/no treatment in adult THA patients?

## 2. Materials and Methods

This systematic review and meta-analysis was conducted according to the Preferred Reporting Items for Systematic reviews and Meta-Analyses (PRISMA) guidelines (Supplementary Materials) [21,22]. The study was registered in the international prospective register of systematic reviews (PROSPERO) with the ID code CRD42024534645.

### 2.1. Inclusion and Exclusion Criteria

A patient intervention compared outcome of interest (PICOS) model was used as follows: in patients undergoing primary hip replacement (P), is the use of bisphosphonates (I) compared to placebo (C) effective in reducing periprosthetic osteolysis (O) according to randomized human controlled trials (S).

Inclusion criteria: human randomised controlled trials. Studies must include periprosthetic bone loss results, as defined by changes in bone mineral density (BMD) over time. All studies must include BMD at baseline (right after the THA operation) and another time point or provide the % change in BMD from baseline. The pharmaceutical must be a bisphosphonate. Exclusion criteria: studies on non-adult patients, animal studies, non RCT studies, in vitro studies, and non-bisphosphonate osteolytic studies. 

### 2.2. Search Strategy

A comprehensive literature search was conducted in four databases (PubMed, Scopus, Embase, and Cochrane) with the following MeSH terms: total AND hip AND (replacement OR arthroplasty) AND (drug OR pharmaceutical) AND osteolysis*.

With the above MeSH terms, we found several studies, and then we looked for duplicate data in publications written by the same research teams and examining the same factors. When two papers were identical, the less detailed one was discarded. Studies were then selected by examining the title and abstract, and then for the remaining studies, a full-text evaluation was performed to determine their eligibility based on the predetermined exclusion and inclusion criteria. As a result, 7 studies were deemed suitable for qualitative analysis [23,24,25,26,27,28,29] (for the exact details of the study selection, please refer to the corresponding next paragraph in the results).

### 2.3. Study Selection

Two independent authors collected the data from the included reports. The reports were fully read, and any data relating to the primary or secondary outcomes were copied. First, tables were searched for the necessary data. If not present, the results were checked as well. A discussion was had for any disagreements on the included data until a consensus was reached. In the case of discrepancy, a third author was consulted.

### 2.4. Data Extraction

**Figure 1:**
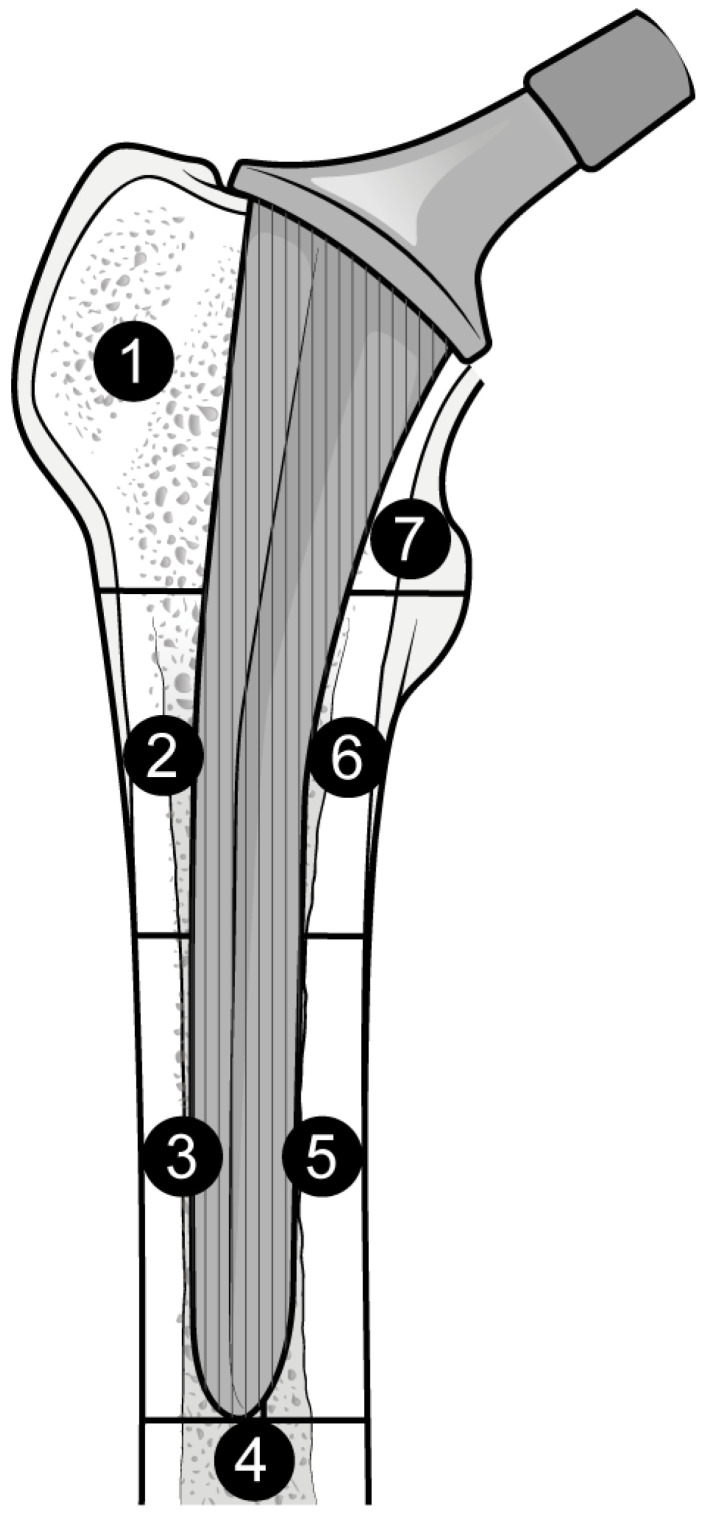
Gruen Femoral zones. Zone 1: greater trochanter. Zone 2: lateral mid stem. Zone 3: lateral distal stem. Zone 4: tip of the stem. Zone 5: medial distal stem Zone 6: medial mid stem. Zone 7: medial calcar.

The outcome for which data was requested was bone mineral density (BMD) changes over time in Gruen’s femoral zones 1 to 7 (Figure 1) [30]. These zones were chosen as they are the primary sites of osteolysis in femoral implants. The secondary outcomes were BMD changes over time in femoral zones 2–6. Therefore, the three most populated time points were selected to perform the meta-analysis: 6, 12, and 24 months. Other variables collected were the type of medicine, gender of participants, mean age, total number of participants, participants in each group, dosing regimen, and duration of pharmaceutical administration.

### 2.5. Risk of Bias

To assess the risk of bias within the included studies, their methodological quality was assessed by using the revised Cochrane risk of bias tool for randomised trials RoB 2 tool; developed by MRC Network of Hubs for Trials Methodology Research (MR/L004933/2-N61), with the support of the host MRC ConDuCT-II Hub (Collaboration and innovation for Difficult and Complex randomised controlled Trials In Invasive procedures—MR/K025643/1), by MRC research grant MR/M025209/1, and by a grant from The Cochrane Collaboration.

### 2.6. Synthesis Methods and Meta-Analysis

The studies were included in each time point (6 months, 12 months, 24 months) according to their availability of data for each time point. Some studies did not have results for all three time points. This may be seen as a “.” where results were unavailable. Some studies required the conversion of summary data units. This was because some studies provided the BMD loss as a ratio from baseline (i.e., subtracting from 100% any BMD loss), while others expressed it directly as a % change. Thus, the former was converted into the latter. For each timepoint, the results were analysed collectively for all Gruen zones and individually for each zone as well to reveal any localized trends and sources of heterogeneity. To assess the effects of bisphosphonates vs. placebo in THA osteolysis on the dichotomous variables, weighted risk ratios (RRs) were calculated to pool the study and control groups in each publication for analysis. Random-effects models were used, regardless of the heterogeneity assessments. Forest plots of the SMD or RRs were generated with all studies. The I2 statistic was used to help assess heterogeneity. Fixed-effects models were used for sensitivity analyses. R Core Team (2023). R: A Language and Environment for Statistical Computing. R Foundation for Statistical Computing, Vienna, Austria. accessed on 1 April 2024 <https://www.R-project.org/>. version 4.2.0 software was used to perform the analyses.

## 3. Results

### 3.1. Prisma Flowchart

**Figure 2:**
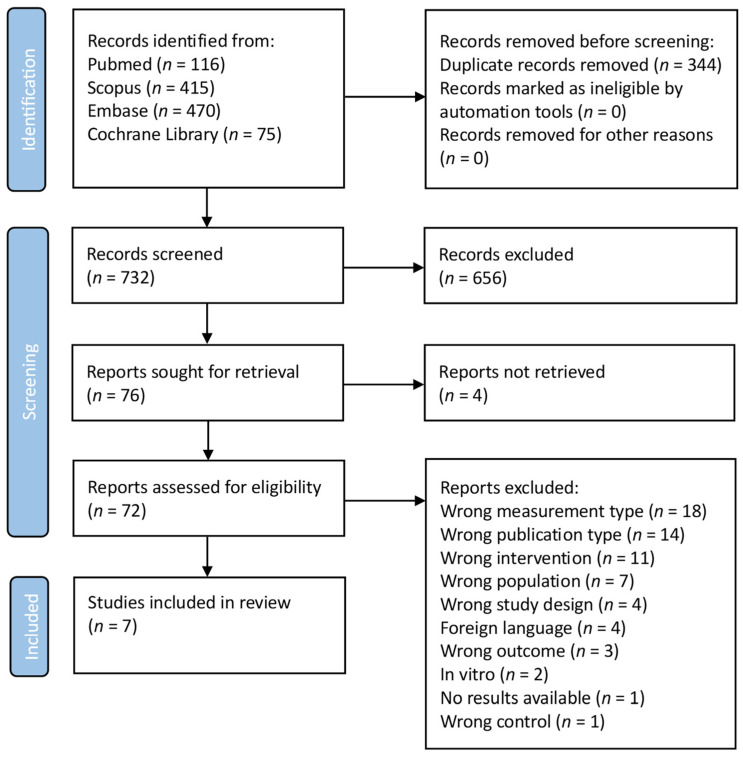
PRISMA flowchart. (Search was performed on 1 March 2023).

As shown in the PRISMA FLOWCHART (Figure 2), 1076 manuscripts were found in the initial search. A total of 344 duplicates were removed before screening. Then, 732 studies were screened and 656 studies were eliminated from consideration based on their title and abstract, because these were deemed unrelated to the current investigation. In total, four reports were not retrieved. To ensure that the remaining 72 manuscripts met the inclusion criteria, a thorough evaluation was conducted: seven studies satisfied the inclusion criteria after full-text screening. The main characteristics of the eligible studies are presented in Table 1.

### 3.2. Risk of Bias Assessment 

**Figure 3:**
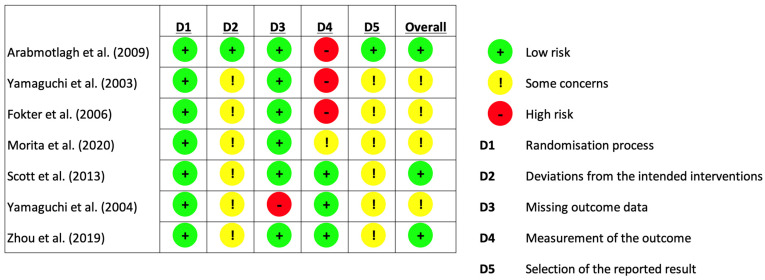
Risk of bias assessment (using Cochrane [31]. Risk of Bias Tool for Randomised Trials).

As seen in Figure 3, the revised Cochrane risk of bias tool for randomised trials (RoB 2 tool) was used in assessing the risk of bias in the included studies [31]. The risk of bias assessment was performed by two independent reviewers. Any discrepancies in their assessment were discussed until a consensus was reached.

### 3.3. Meta-Analysis

**Figure 4:**
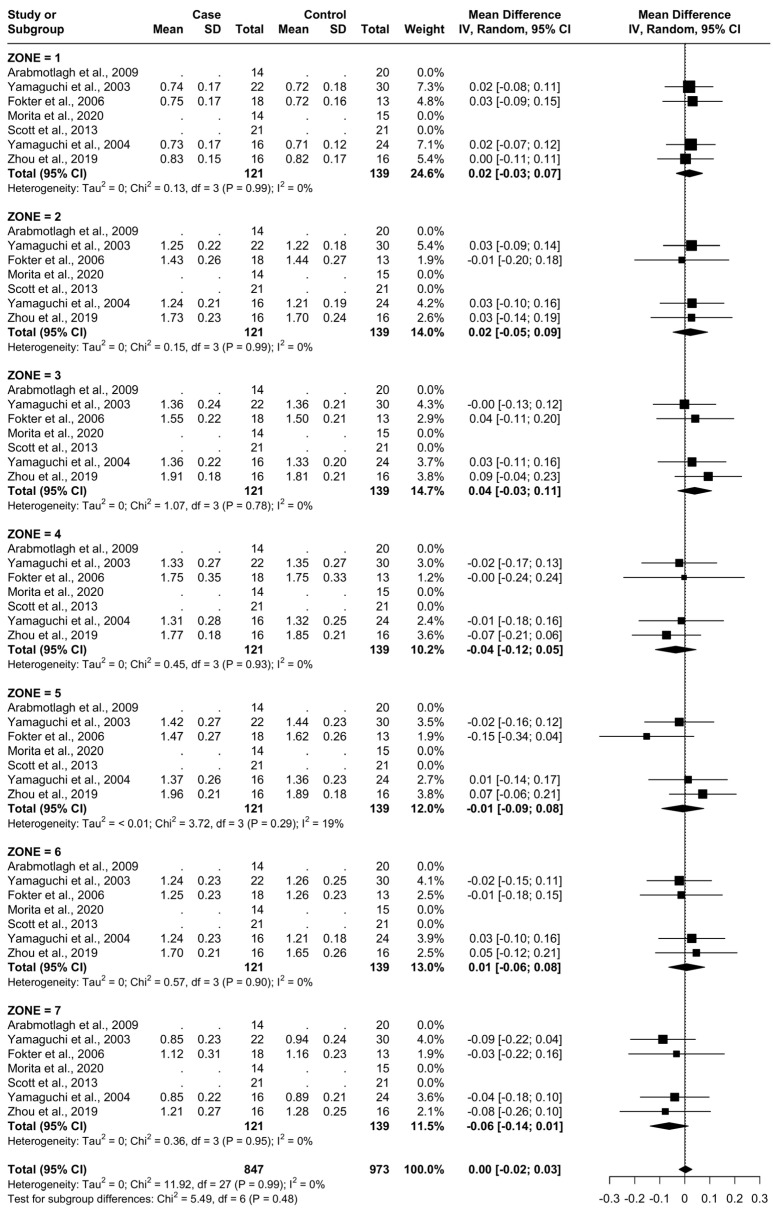
Baseline characteristics of patients.

For all zones, there were a total of 121 patients being administered a clinically effective dose of a bisphosphonate (named as “Case“) and 139 being administered a placebo or no treatment (named as “Control“). The specific treatments administered in each study are presented in Table 1. The BMD at baseline time 0 for each femoral Gruen’s zone can be seen in Figure 4.

### 3.4. Results at 6 Months 

**Figure 5:**
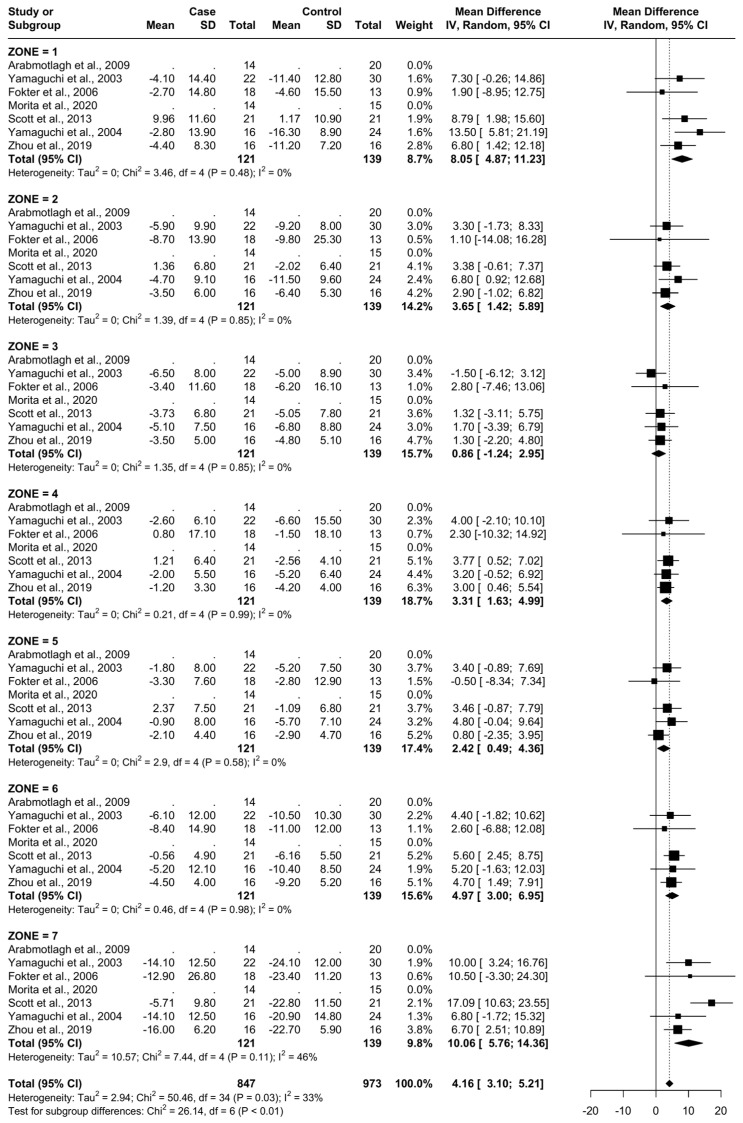
Results at 6 months.

**Figure 6:**
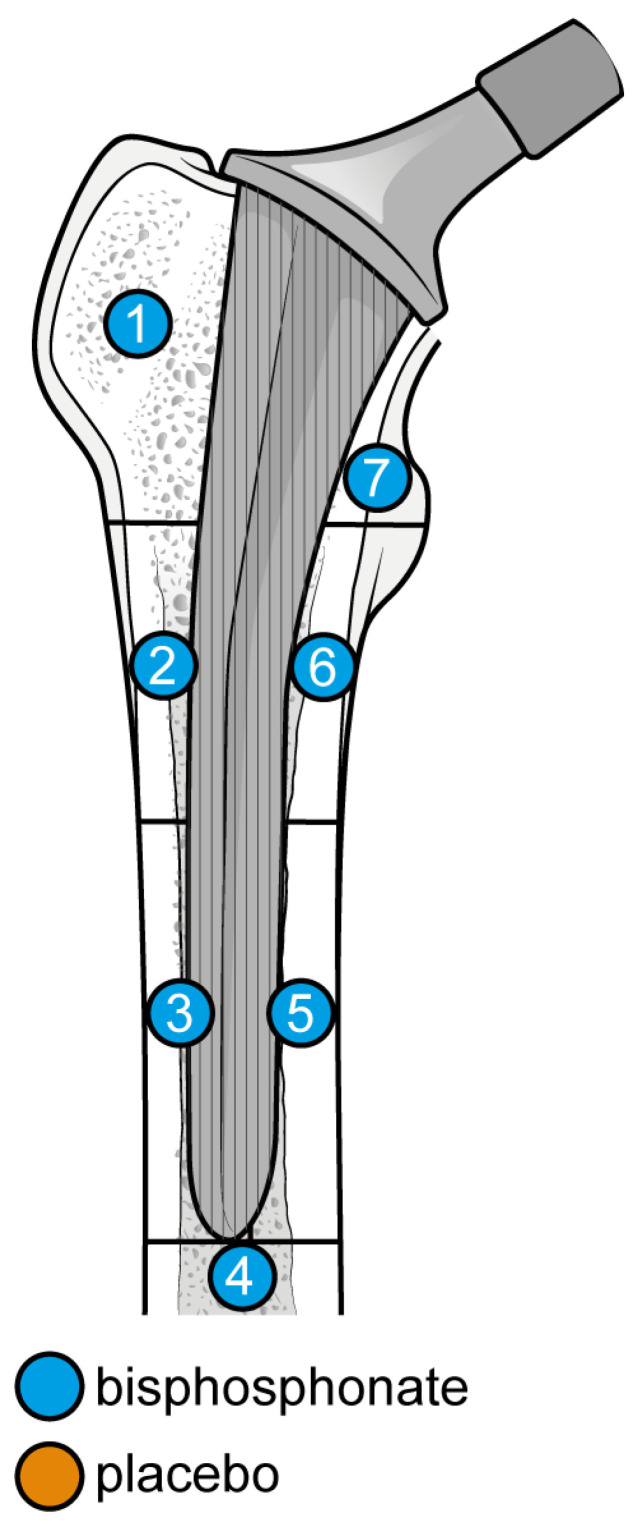
Graphical representation of the results illustrated in Figure 4 by forest plot (coloured dots: favour of bisphosphonates or placebo in reducing BMD loss divided by Gruen femoral zones).

From baseline to 6 months, five studies were included. A total of 93 patients were administered bisphosphonates and a total of 104 people were administered a placebo or no treatment. There was a low heterogeneity (I^2^ = 33%). The Standard Mean Difference (SMD) using the random model was 4.16, 95% Confidence Interval (CI) [3.10, 5.21], *p* < 0.01. In all Gruen’s femoral zones, the data were in favour of bisphosphonates, with a peak SMD of 10.06, 95% CI [5.76; 14.36] in zone 7 and 8.05, 95% CI [4.87; 11.23] in zone 1. (Figure 5 and Figure 6).

### 3.5. Results at 12 Months

**Figure 7:**
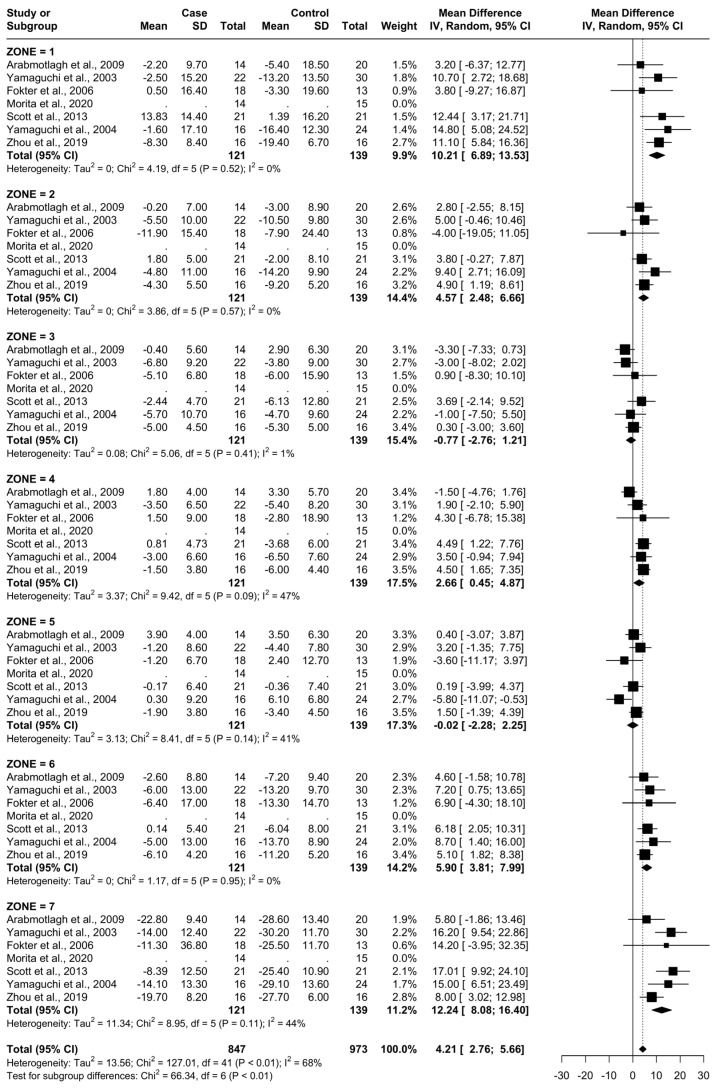
Results at 12 months.

**Figure 8:**
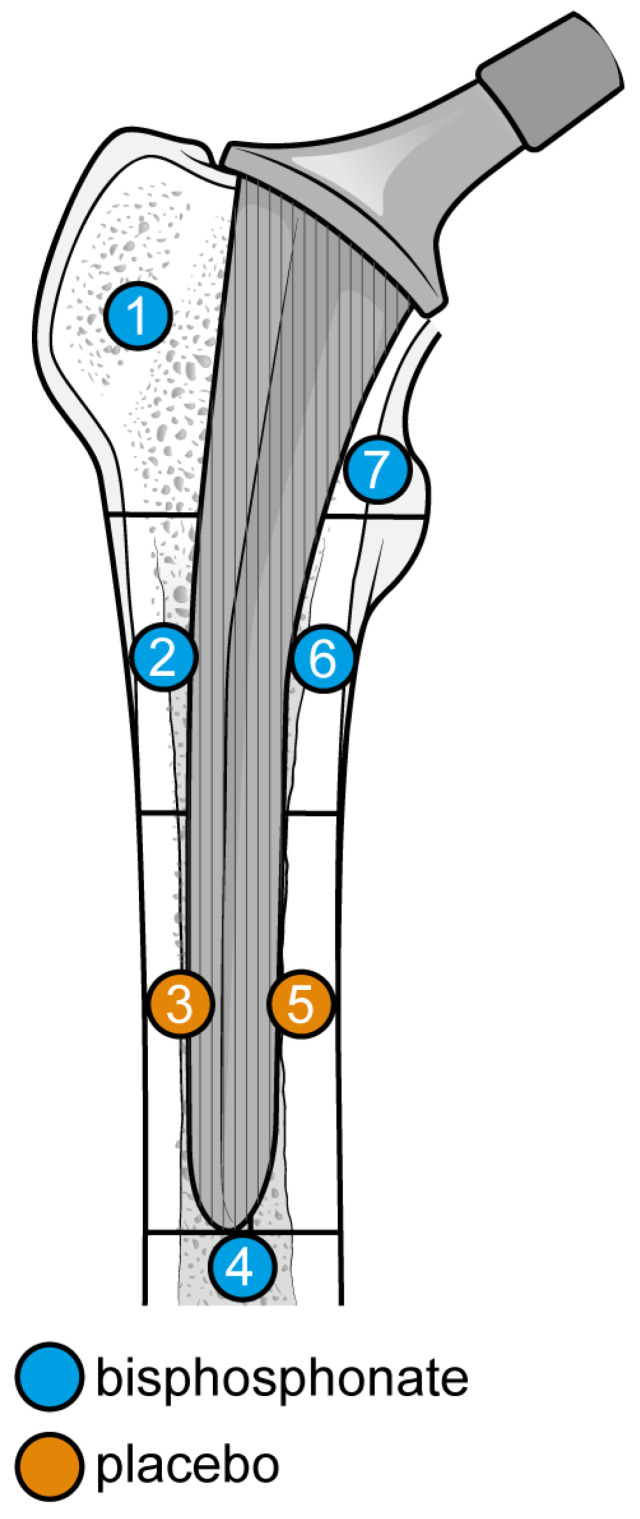
Graphical representation of results illustrated in Figure 6 by forest plot (coloured dots: favour of bisphosphonates or placebo in reducing BMD loss divided by Gruen femoral zones).

From baseline to 12 months, six studies were included. A total of 107 patients were administered bisphosphonates and a total of 124 people were administered a placebo or no treatment. There was a high heterogeneity (I^2^ = 68%). The SMD using the random model was 4.21, 95% CI [2.76, 5.66], *p* < 0.01. In Gruen’s femoral zones 1, 2, 4, 6, and 7, the data were in favour of bisphosphonates, with a peak SMD of 12.24, 95% CI [8.08; 16.40] in zone 7 and 10.21, 95% CI [6.89; 13.53] in zone 1. In zones 3 and 5, the data were in favour of placebo/no treatment (Figure 7 and Figure 8).

### 3.6. Results at 24 Months

**Figure 9:**
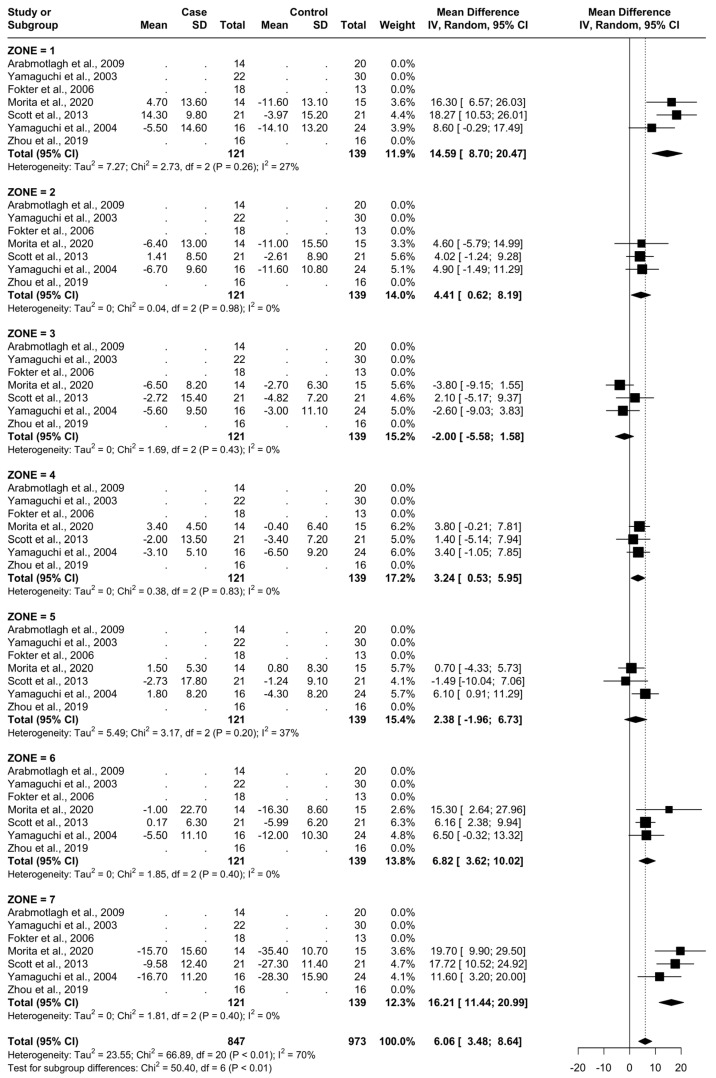
Results at 24 months.

**Figure 10:**
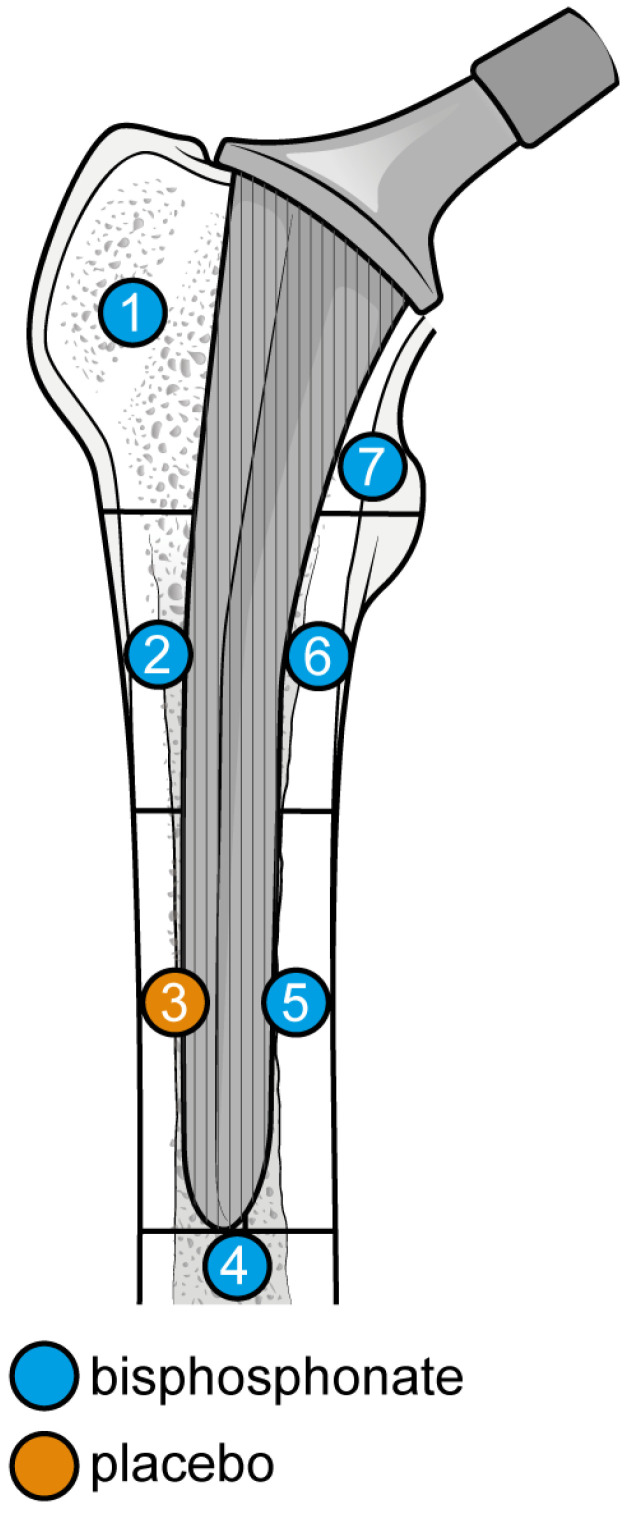
Graphical representation of results illustrated in Figure 8 by forest plot (coloured dots: favour of bisphosphonates or placebo in reducing BMD loss divided by Gruen femoral zones.

From baseline to 24 months, three studies were included, as seen in Figure 8. A total of 51 patients were administered bisphosphonates and a total of 60 people were administered a placebo or no treatment. There was a high heterogeneity (I^2^ = 70%). The SMD using the random model was 6.06, 95% CI [3.48, 8.64], *p* < 0.01. In Gruen’s femoral zones 1, 2, 4, 5, 6, and 7, the data were in favour of bisphosphonates, with peak a SMD of 16.21, 95% CI [11.44; 20.99] in zone 7 and 14.59, 95% CI [8.70; 20.47] in zone 1. In zone 3, the data were in favour of placebo/no treatment with an SMD of −2.00, 95% CI [−5.58; 1.58] (Figure 9 and Figure 10).

## 4. Discussion

At 6 months, the most significant differences in the reduction in BMD consequent to the usage of bisphosphonates were noticed in zones 1, 6, and 7 (random SMDs of 8.05, 4.9, and 10.06, respectively). Heterogeneity was low in all these areas except 7, where it was moderate.

At 12 months, the most significant differences were again noted at zones 1, 6, and 7 with the addition of zone 2 (random SMDs of 10.21, 5.90, 12.24, and 4.57 respectively). A low heterogeneity was observed in zones 1, 2, 3, and 6. A moderate heterogeneity was observed in zones 4, 5, and 7. At 24 months, the most significant difference was observed in zones 1, 6, and 7 (random SMDs of 14.59, 6.82, and 17.21, respectively). Heterogeneity was low in zones 2, 3, 4, 6, and 7. Heterogeneity was moderate in zones 1 and 5. 

This is in line with the knowledge that bisphosphonates are selectively internalised by osteoclasts and disrupt their normal function [32], causing apoptosis, as bone resorption was significantly reduced in the 6-to-24-month period. Also of interest is that the highest results were noted in regions 1 and 7 in all three time points. This is logical, as these are the zones closest to the stem and have the highest potential for friction with the prosthesis, leading to resorption. Therefore, the highest difference between the study and control group would be seen in this area, as it is the region with the highest capacity for a reduction in resorptive effects. 

A meta-analysis on zoledronic acid used in 6 studies with 307 patients found it to be efficacious for the treatment of BMD loss after a hip arthroplasty; there was significantly lower BMD loss at 6 and 12 months vs. controls. At 6 months, the zoledronic acid groups were found to have significantly less BMD loss vs. controls in Gruen zones 1, 2, 4, 6, and 7. At 12 months, they had a significant difference vs. controls in zones 1, 2, 4, 5, 6, and 7 [33]. Another meta-analysis on four studies with 185 patients found that a significant difference was observed vs. controls in zones 1, 2, 4, 6, and 7 between 6 and 24 months [34]. Thus, it appears that zoledronic acid is, in fact, efficacious between 6 and 24 months. This agrees with the findings of Zhou et al. and Scott et al. in our study. A network meta-analysis including etidronate found it to be efficacious at 6 months (SMD 0.42, 95% CI 0.09 to 0.76) and 12 months (SMD 0.75 95% CI 0.42 to 1.07) [35]. Another study found that the efficacy of etidronate vs. controls at 6 months was 9.2% in zone 1 (*p* < 0.001) and 8% in zone 7 (*p* = 0.003). At 12 months, the etidronate group displayed a 7.2% higher BMD than controls in zone 1 (*p* < 0.001). Zone 7 did not display any significant difference. At 24 months, neither zone had a significant result between the study and control groups [36]. A last study suggested that there was, in fact, no significant difference between etidronate and placebo at 6 or 12 months and, thus, suggested that other bisphosphonates be used [25]. The studies of Yamaguchi et al. and Fokter et al. displayed a high efficacy at all time points. This may slightly deviate from the assumption that the efficacy would drop between 12 and 24 months based on the network meta-analysis [36].

For alendronate, a randomised control trial of 48 patients found that alendronate-treated patients displayed a significant difference at zone 7 at 49 weeks vs. control (*p* = 0.0384), but not at 6 nor 12 months [25]. The network meta-analysis cited earlier found it did not have a significant effect versus controls, neither at 6 months nor at 12 months [35]. Considering the findings of Arabmotlagh et al., its efficacy remains unclear. However, the network meta-analysis found it to be more efficacious when paired with alfacalcidol. For the treatment of bone loss after total hip arthroplasty (THA), there are other drugs that can be reported for full disclosure, including monoclonal antibodies like denosumab, romosozumab, teriparatide, a synthetic variant of the parathyroid hormone, and raloxifene, a selective oestrogen modulator. Denosumab, a monoclonal antibody, blocks osteoclast formation by binding to RANKL [25]. Studies suggest that it is significantly effective in preventing bone mineral density (BMD) loss in Gruen zone 7 at one year and has noticeable effects up to 24 months [37]. The difference decreased over time to 15% at 24 months after surgery in zone 7 and 4% in all zones. To compare with our findings, the denosumab effect seems to be similar up to 12 months. However, in bisphosphonates, it was noticed that the mean difference in zone 7 became more pronounced in the 12-to-24-month period.

Teriparatide, a synthetic parathyroid hormone derivative, had a prophylactic effect on BMD in Gruen zone 7 after 12 months. When used daily, it results in greater BMD at the femoral neck and total hip compared to placebo [38]. Compared to our study, teriparatide appears to be equally efficacious; the difference versus control for bisphosphonates followed a similar pattern of efficacy in both 12 and 24 months. This is further supported by the meta-analysis cited above, which found no significant difference between the efficacy of teriparatide and bisphosphonates.

Raloxifene, a selective oestrogen modulator, has anti-resorptive properties in female THA patients. At 6, 12, and 24 months, daily doses result in much less bone loss than the control group, particularly in zones 1 and 7 [39]. While research on its involvement in periprosthetic osteolysis is limited, it appears to be promising, particularly in postmenopausal women. In conclusion, these comparative medications have variable efficacies in treating periprosthetic bone loss, showing promise in certain patient categories and timeframes.

### Limitations and Implications

Differences in the study protocols between RCTs led to high heterogeneity in some groups. Doses were often taken at a certain interval that was not consistent between studies. Some of the RCTs had a moderate risk of bias, making their results less reliable. Large RCTs were also lacking. Another limitation is that there were not enough studies to perform a subgroup analysis of cementless versus cemented THA and how it may affect the efficacy of bisphosphonate treatment. Finally, some potentially useful studies had to be excluded due to differences in the measurement parameters (BMD ratio vs. percent change of BMD and standard deviation vs. CI). The meta-analysis reconfirmed the efficacy of bisphosphonates in the 6–12-month timeframe using the most recent applicable studies. It also provided evidence for the 12–24-month timeframe, which has not been re-evaluated in meta-analyses in over a decade. This study is, thus, useful in reaffirming the efficacy of bisphosphonates given more recent data. Further research is, therefore, justified. Future studies should be focused on increasing group sizes and collecting results beyond the 2-year mark. Research should also be focused on examining the potential of female-exclusive post-menopausal antiresorptive treatments (raloxifene and calcitonin) in combination with bisphosphonates for any evidence of increased efficacy.

## 5. Conclusions

Bisphosphonates decrease the periprosthetic osteolysis attained after a hip replacement. Evidence from seven RCTs demonstrated a significant difference between bisphosphonates and controls when it comes to BMD loss over time. Specifically, bisphosphonates outperformed controls (placebo or no treatment) in the 6-to-24-month period post-operation. The zones nearest to the site of the hip stem (Gruen Zones 1 and 7) displayed the largest difference when compared to controls. The data analysis demonstrated that the usage of bisphosphonates is linked to a significant decrease in bone mineral density loss, indicating a potential medium-term protective effect on implant integrity. Future studies should address the limitations of the included studies, which include the relatively small sample sizes and the restricted duration of follow-up. To confirm these initial findings and evaluate the long-term efficacy of bisphosphonates in avoiding periprosthetic osteolysis, future research should concentrate on larger trials with longer follow-up periods. Furthermore, it would be interesting to investigate the possible combinations of bisphosphonates with other medications to improve treatment efficacy and offer broader protection against osteolysis and BMD loss. Improved clinical results and the durability of hip prosthetic implants will be the ultimate goals of these research, which will also lower the risk of surgical revision and improve the quality of life for patients undergoing hip replacements.

## Figures and Tables

**Table 1:** Study characteristics.

Author	Year	Bis. Dose	Cont. dose	Bis. *n*	Cont. *n.*	Follow-Up (Months)
Arabmotlagh et al. [23]	2009	10 mg/day alendronate for 5 weeks	No treatment	14	20	12 months
Yamaguchi et al. [24]	2003	400 mg/day etidronate was given in a 2-week cycle followed by 12 weeks of 500 mg/day calcium supplementation	No treatment	22	30	12 months
Fokter et al. [25]	2006	400 mg etidronate/day, given in a 2-week cycle followed by 12 weeks of 260/mg/day calcium supplementation	Placebo	18	13	12 months
Morita et al. [26]	2020	20 μg/day teriparatide for 1 year. Patients then switched to 35/mg/week alendronate for 1 year	No treatment	14	15	24 months
Scott et al. [27]	2013	IV infusion of 5 mg zoledronic (+received oral calcium carbonate 1200 mg/day and calcitriol 0.50 μg/day)	Oral calcium carbonate 1200 mg/day and calcitriol 0.50 μg/day	21	21	24 months
Yamaguchi et al. [28]	2004	Received 400 mg/day of etidronate for 2 weeks, followed by 500 mg/day of calcium for 12 weeks. Cycle was repeated every 14 weeks for four cycles for a total of 12 months	Placebo	16	24	24 months
Zhou et al. [29]	2019	Received an intravenous infusion of 5 mg zoledronic acid (+1200 mg/day calcium carbonate and 0.50 μg/day calcitriol).	1200 mg/day calcium carbonate and 0.50 μg/day calcitriol	21	21	24 months

## Supplementary Materials

The following supporting information can be downloaded at: https://www.mdpi.com/article/10.3390/biomedicines12081778/s1, PRISMA 2020 Main Checklist.
